# Genotype by environment interaction effect on some selected traits of orange-fleshed sweet potato (*Ipomoea batatas* [L].Lam)

**DOI:** 10.1016/j.heliyon.2022.e12395

**Published:** 2022-12-17

**Authors:** Getachew Etana Gemechu, Tewodros Mulualem, Neim Semman

**Affiliations:** a Department of Horticultural Crop Research, Crop Science Research Process, Jimma Agricultural Research Center, P.O. Box. 192, Jimma, Ethiopia; b Ethiopian Institute of Agricultural Research, P.O. Box. 2003, Addis Ababa, Ethiopia

**Keywords:** AMMI analysis, Environment, Genotype, GGE-Biplot, Yield, Aboveground biomass weight, Storage root girth, Storage root length, Stability, Total storage root number, Variations

## Abstract

In Ethiopia, sweet potato is the 2nd and the most important root crop after Enset [*Ensete ventricosum* (Welw) Cheesman]. Even though widely cultivated in Ethiopia, the occurrences of wide agro-ecological variability are the key challenge for the selection of high yield and stable orange-fleshed sweet potato genotypes due the high interaction of genotype by environment effect (GEI). Until to date, the research reports on GEI and stability of orange-fleshed sweet potato genotypes under Southwest and West of Ethiopian conditions is very limited. Due to this fact, the study was conducted in main sweet potato growing of Southwest and West of Ethiopia since the 2019–2020 growing seasons. Nine Orange-fleshed sweet potato genotypes were evaluated in four different ecologies in completely randomized block design with three replications. The mainly indicative data and related variables were collected and analyzed by using ANOVA, AMMI, and GGE biplots. The ANOVA for total storage root yield revealed significant difference in the environments, genotypes, and the interactions of both (P < 0.001). The genotypes NASPOT-12 was the mostly performed one over all the rest with total fresh storage root yield of 55.88 ton ha^−1^ while Kulfo was performed as the lowest. The AMMI1 and AMMI2 biplot analysis revealed that, the genotypes NASPOT-12 was resulted in an above average mean of total fresh storage root yield and the genotype Koka-12, NASPOT-13, NASPOT-12, and Kulfo were far apart from the biplot origin which indicated the genotypes were the more responsive and largely contributed to the interaction component and thus considered as specifically adapted genotypes respectively. The GGE biplot revealed that the total variations of 79% with the PC1 accounted about 55% and the PC2 affect about 24% of variations approximately on the total fresh storage root yield of the tested orange-fleshed sweet potato genotypes. The “which won where” identified the three growing mega environments. In the comparison and ranking of the genotypes by the GGE biplot analyses, the genotype NASPOT-12 was more desirable than the rest genotypes. In the comparison and ranking of the environments by this analysis, the environment Jimma-1 was the most representative followed by Agaro-2 in the total fresh storage root yield of orange-fleshed sweet potato genotypes. Above all the genotype NASPOT-12 was responded well in most of the environments hence, recommended genotype for multipurpose advantage.

## 1 Introduction

Sweet potato [*Ipomoea batatas* (L) Lam] is among the most important crop of root and tuber crops which belongs to the family Convolvulaceae. It was originated from the central tropical America which was cultivated at least 5000 years ago (Ahn, 1993). The crop spread was too early throughout the regions including the Caribbean which currently known as the southern United States (Zhang et al., 1998). Historically it brought to the Europe by the Spanish and Portuguese explorers and then its cultivation quickly spread over the old world up to the African continent (Woolf, 1992). Currently, it has been cultivated in different areas of tropical to sub-tropical region of the world (Gemechu et al., 2020). It plays a significant role as a food security crop, economical, and income generation in sub-Saharan Africa. In other case, as compared to other root crops like taro (Kifle et al., 2020), sweet potato has an advantage of higher yield potential and adapted to wide range of ecologies and also well adapted to drought affected ecologies. Further, the crop is a source of vitamin A that serves in the prevention of vitamin A deficiency-related health problems (Gurmu et al., 2015; Manrique and Hermann, 2000).

In Ethiopia, sweet potato is the 2^nd^ and the most important root crop after Enset [*Ensete ventricosum* (Welw) Cheesman] (CSA, 2020). It is widely grown in wide range in Ethiopia, typically in southern and Oromia region for food, feed, and economic income (Gurmu et al., 2015). Even though, they adapted to wide range of environments, sweet potato varieties genotypes are/were evaluated for their yield and yield related traits in different agro ecologies and they may/were resulted in wide differences in yield performances of the genotypes over environments. The occurrences of wide agro-ecological variability are the key challenge for the stable sweet potato genotype selection due the high interaction of genotype by environment effect (GEI).

Several studies have been conducted on genotype by environment interaction (GEI) and the stability of sweet potato germplasm under various ecological conditions on sweet potato and other crops (Yan, 2002; Duma et al., 2019). For instance Ebem et al. (2021) stated that in order to target the breeding program, GEI is necessitated to estimate the magnitude of genotype × environment interaction (G × E) and to select stable and high yielding sweet potato genotypes for fresh root yield and other important traits in multiplications in order to identify the most discriminating and representative test environments. Similar result was reported from south Ethiopian region on GEI of sweet potato genotypes(Gurmu et al., 2017)Until to date, the report on GEI and stability of orange flashed sweet potato genotypes under Southwest and West of Ethiopian conditions is very limited. Such information on this stated crop genotypes across the ecology of southwest area of Ethiopia will provide scientific bases to develop new generally and specifically adaptable genotypes in the future breeding strategies for the target ecology. Therefore the GEI and stability analysis are the crucial one for such unique studies in breeding programs. To undertake these, several statistical models are available to quantify the extent of the genotypes. The Additive main effect and Multiplicative Interaction (AMMI) (Gauch and Zobel, 1996; Caliskan et al., 2007; Gauch, 2013) and Genotype plus Genotype by Environment Interaction (GGE biplot) (Yan et al., 2000; Yan et al., 2001; Yan, 2002; Duma et al., 2019) are the most commonly widely used models for analyzing Multi-environment data of the crop yield and related trait. Farther more Caliskan et al. (2007) stated that the AMMI model appeared to be a better option for evaluating both GE interaction and stability of sweet potato genotype in Multiplication trials. Therefore this study was designed to assess the nature and the extent of the GEI and advance insights into mega-environments for introduced orange fleshed sweet potato genotypes under the condition of southwest Ethiopia.

## 2 Materials and methods

### 2.1 Experimental location descriptions

**Figure 1:**
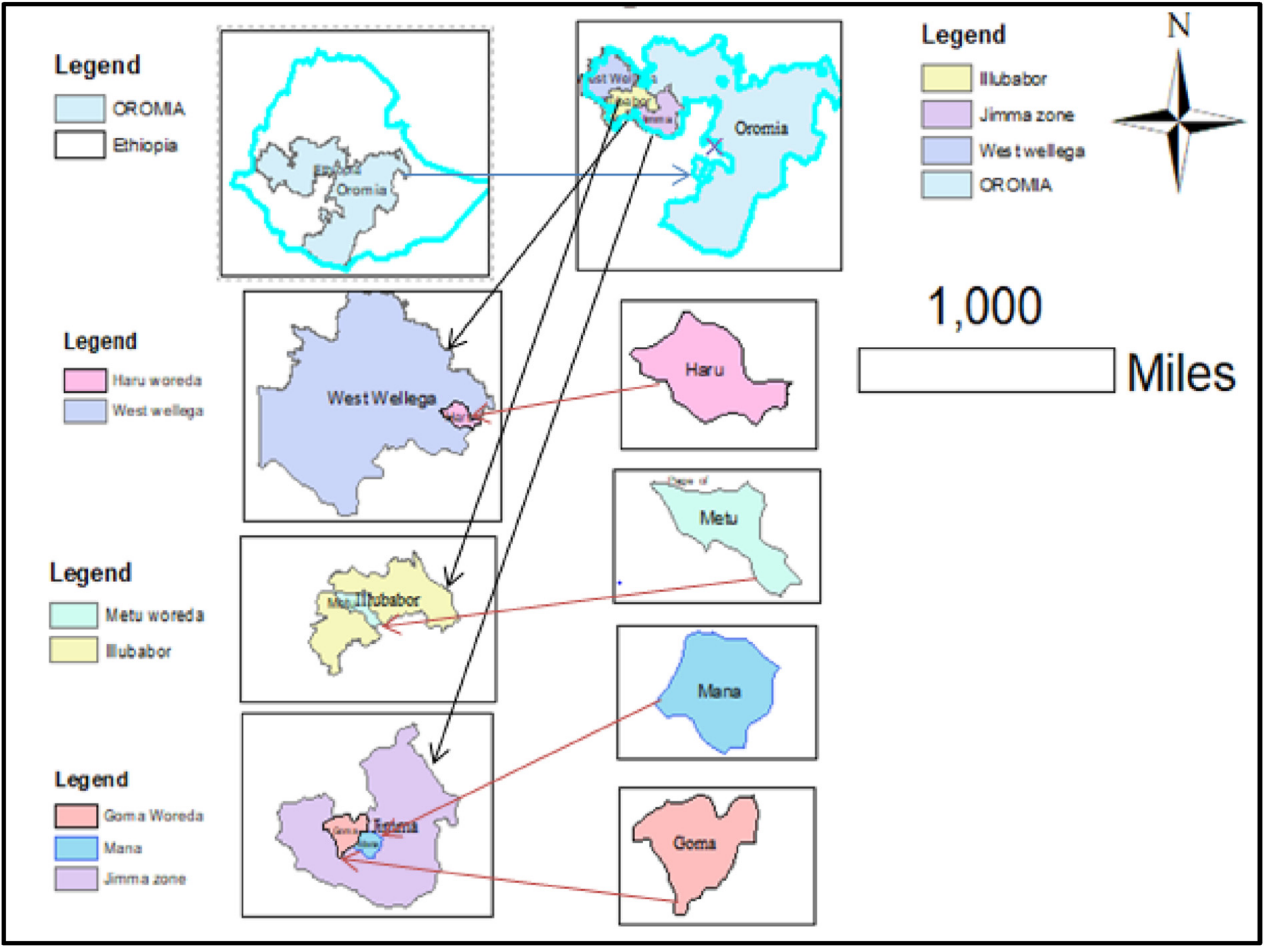
Map of the experimental locations.

The field experiment was conducted in four testing locations namely: Jimma, Agaro, Metu, and Haru. Jimma is one among research centers found under Ethiopian Institute of Agricultural Research and the rest are the sub-centers of Jimma agricultural research centers. They are/were considered as the representative location for sweet potato growing areas of Southwest and West Ethiopia. The experiment was conducted for two cropping seasons/years (2019–2020) in all four locations which made a total of eight environments considering one location and one cropping season as one environment. The description of the location, agro-ecological, and climatological conditions of the study sites is summarized in Figure 1 and Table 1 respectively.

**Table 1:** The summary of the agro-ecological and climatic description of the study areas.

Location	Altitude (m.a.s.l.)	Latitude	Longitude	Rainfall (mm)	Temperature (°C)	
					Maximum	Minimum
Jimma	1753	7° 40.00′ N	36° 47′.00′ E	1521.1	26.2	12.1
Agaro	1560	7°51′ .00′ N	36°51′ 35′ E	1520	23.35	12.6
Metu	1550	8°18′ .00′ N	35°35′ .00′ E	1520	28.0	12.2
Haru	1750	8°58′00′ N	38°48′00′ E	1727	21.5	12.2

### 2.2 Experimental materials and design

A total of nine orange-fleshed sweet potato varieties/genotypes were evaluated for their yield and related performance as well as their stability under rain fed at different testing sites (Table.2). The experiment was laid out in a randomized completely block design (RCBD) with three replications. The RCBD was selected because of that the test environment are located at different agro ecological condition some of the having different bimodal rain falls and soil types. These variations, the slope of the test site and the number of the genotypes determined us to use the design stated.

**Table 2:** List of tested genotypes/varieties.

Treatment No	Genotypes	Origin
1	Alamura	Ethiopia
2	Dilla	Ethiopia
3	Guntutei	Ethiopia
4	Kabode	Uganda
5	Koka-12	Ethiopia
6	Kulfo	Ethiopia
7	NASPOT-12	Uganda
8	NASPOT-13	Uganda
9	VITA	Uganda

### 2.3 Experimental procedures

The land was plowed twice during the dry season to reduce weed and insect pest infestation before planting at all locations. During planting land was harrowed, mowed, softened and ridges were prepared and the cutting planting materials were planted on the ridge. Recommended Intra and interspacing of 30 cm and 60 cm were used. A total of 1.5 × 2.4 (3.6 m^2^) plot size with five rows per plot was used accommodating 25 plants per plot.

### 2.4 Data collection

Data were collected from ten (10) plants from each plot and the average values were used for data analysis. The characters that are used for data collection were: vine length (cm), marketable storage root number, storage root length (cm) storage root girth (mm), the weight of above-ground fresh biomass (ton ha^−1^), total fresh storage root weight (ton ha^−1^) and harvest index (%). The number of marketable storage roots represents the number of roots that were more than or equal to 100 g (Levette, 1993) or with diameters at the widest point >25 mm roots. These were counted and the number recorded per plot. Number of unmarketable roots represents the number of roots that are <100 g or 25 mm at the widest point (Levette, 1993; Stathers et al., 2003). Weight of marketable roots is the weight (kg/plot) of roots suitable for marketing. Weight of unmarketable roots is the weight (kg/plot) of roots not suitable for marketing. Total fresh root yield (ton ha^−1^) weight is obtained as the sum of weights of marketable and unmarketable roots converted to tons per hectare.

### 2.5 Data analysis

Homogeneity of residual variance was tested before combined analysis over locations in each year as well as over locations and years (for the combined data) using Bartlet’s test (Steel and Torrie, 1986). Accordingly, the data collected indicated homogenous variance. A normality test was also conducted and all data showed normal distribution. A combined analysis of variance was performed using GenStat 14th edition (Payne et al., 2011), Statistical Analysis Software (ce:cross-ref>SAS Institute Inc, 2000), and SAS version 9.0 (Yuan, 2010) statistical software. Treatment means were separated by using the Fisher’s protected least significant difference (LSD) test at 1% and 5% probability. The model employed in the analysis was;

Y_ijk_ = μ+G_i_ + E_j_ + B_k_ + GE_ij_ + ϵ_ijk_

where: Y_ijk_ is the observed mean of the i^th^ genotype (G_i_) in the j^th^ environment (E_j_), in the K^th^ block (B_k_); μ is the overall mean; G_i_ is an effect of the i^th^ genotype; E_j_ is an effect of the j^th^ environment; B_k_ is block effect of the i^th^ genotype in the j^th^ environment; GE_ij_ is the interaction effects of the i^th^ genotype and the j^th^ environment, and ϵ_ijk_ is the error term.

AMMI and AMMI biplot analysis, showing the genotype and environment mean against Interaction Principal Component analysis one (IPCA1), and Interaction Principal Component analysis one (IPCA1) against Interaction Principal Component analysis two (IPCA2) were also performed using Meta-analysis procedure-I using the same statistical software. GGE biplot was also executed using the Meta-analysis of GenStat 17^th^ edition.

## 3 Results and discussions

### 3.1 Combined analysis of variance and estimation of variance component

The results revealed from the analysis of variance of all the evaluated traits and genotypes are illustrated in (Table 3). The genotype, environment, and GEI variance were analyzed to reveal the overall performance of the evaluated genotype and related traits. Accordingly they showed that a highly significant variation (p < 0.001) in analysis of all the evaluated orange fleshed sweet potato traits. In case of the genetic variability, the ANOVA (Table 3) also showed that the ecologies in which the experiment was conducted were different from one another in their responding the tested orange fleshed sweet potato genotypes. Likewise, it also revealed that the responses of the genotypes were tottering in the expression of their traits with change in the ecology.

**Table 3:** Mean squares for yield and yield related traits of orange fleshed sweet potato genotypes across ecologies.

Sources of variation	DF	Mean squares						
		TSRW	VL	SRL	SRG	MSRN	WAGB	HI
Block	16	118	1378.0	12.02	126.8	0.38	163.0	93.1
Genotype (G)	8	426∗∗∗	14612.0∗∗∗	56.06∗∗∗	537.4∗∗∗	3.26∗∗∗	1462.0∗∗∗	149.9∗∗∗
Environment (E)	7	11632∗∗∗	18549.0∗∗∗	154.2∗∗∗	4057.1∗∗∗	11.08∗∗∗	7747.0∗∗∗	1443.6∗∗∗
G∗E	56	154∗∗∗	1169.0∗∗∗	8.72∗	125.0∗∗∗	0.84∗∗∗	618.0∗∗∗	136.2∗∗∗
Residual	30	78	598.0	4.73	58.4	0.41	254	53.0

∗, ∗∗, ∗∗∗ significant at 0.05, 0.01, and 0.001% probability level.

DF = Degree of freedom, VL = Vine length (cm), SRL = Storage root length (cm), SRG = Storage root girth (mm), MSRN = Marketable storage root number per plant, WAGB = weight of above ground bio mass (ton ha^−1^), TSRW = Total fresh storage root weight (ton ha^−1^), HI = harvestable index (%).

Regarding contribution to the variability concerned, most of the traits' contribution to environmental variance was higher (ranging from 36.87% for vine length to 83.35% for total fresh storage root yield) and followed by GEI and genotype respectively (Table 4). An anonymous result was reported by Berihun et al. (2019); Gurmu and Mekonen (2019) on Irish potato and sweet potato. Concerning total fresh storage root yield, the most source of variance was typically the inherent genetic made up which is simply the genetic effect (33.19%) (Table 4). This had a similarity with the result reported by Gurmu et al. (2015).

**Table 4:** The combined sum of squares for yield and related variables of orange fleshed sweet Potato genotypes evaluated during the 2019–2020 cropping season.

Sources of Variation	DF	TSRW	VL	SRL	SRG	MSRN	WAGB	HI
Block	16	1888 (1.93)	22048 (6.26)	192.32 (8.18)	2028.8 (4.67)	6.08 (3.6)	2608 (2.35)	1489.6 (6.77)
Gen	8	3408 (3.49)	116896 (33.19)	448.48 (19.08)	4299.2 (9.89)	26.08 (15.43)	11696 (10.56)	1199.2 (5.45)
Env	7	81424 (83.35)	129843 (36.87)	1079.4 (45.92)	28399.7 (65.32)	77.56 (45.88)	54229 (48.96)	10105.2 (45.91)
Gen∗Env	56	8624 (8.83)	65464 (18.59)	488.32 (20.78)	7000 (16.10)	47.04 (27.82)	34608 (31.25)	7627.2 (34.65)
Residual	30	2340 (2.40)	17940 (5.09)	141.9 (6.04)	1752 (4.03)	12.3 (7.28)	7620 (6.88)	1590 (7.22)
Total	117	97684	352191	2350.42	43479.7	169.06	110761	22011.2

*Note:* Numbers inside and outside parenthesis was SS and % of SS of traits, respectively. DF = Degree of freedom, Gen = Genotype, Env = Environment, TSRW = Total fresh storage root weight (ton ha^−1^), VL = Vine length (cm), SRL = Storage root length (cm), SRG = Storage root girth (mm), MSRN = Marketable storage root number per plant, WAGB = weight of above ground fresh biomass (ton ha^−1^), HI = Harvestable index (%).

### 3.2 The yield and related variable performance of the orange fleshed sweet potato genotypes

An average total fresh storage root yield of the evaluated orange-fleshed sweet potato genotypes over the eight environments was 45.45 ton ha^−1^. NASPOT-12 was resulted in the highest average total fresh storage root yield (55.88 ton ha^−1^), followed by NASPOT-13 (47.55 ton ha^−1^) while Kulfo resulted in the lowest yielding genotypes (42.39 ton ha^−1^) (Table 5). Similar to this, Ebem et al. (2021) reported that the variation of the genotypes in his evaluation of 41 sweet potato genotypes in two different locations. The genotype NASPOT-12 had the highest average storage root girth (71.53 mm), marketable storage root number (2.93), and weight of above ground fresh biomass (61.50 ton ha^−1^). While, Alamura, Kabode, and Kulfo produced the lowest storage girth, marketable storage roots number, and weight of above ground fresh biomass, respectively.

**Table 5:** A combined mean yield and related variables of Orange fleshed sweet potato genotypes across the eight tested environments.

Genotypes	TSRY	VL	SRL	SRG	MSRN	WAGB	HI
Alamura	43.91^bc^	147.02^b^	18.82^ab^	58.80^b^	2.95^a^	50.66^b^	0.46^c^
Dilla	44.31^bc^	167.02^a^	18.98^ab^	60.29^b^	2.89^a^	47.96^bc^	0.48^cb^
Guntutei	43.04^bc^	108.3^cd^	20.12^a^	59.41^b^	2.31^bc^	43.11^bcd^	0.49^bc^
Kabode	42.90^bc^	103.1^d^	19.22^ab^	62.11^b^	2.01^c^	38.58^d^	0.51^ab^
Koka-12	45.55^bc^	156.95^ab^	17.99^bc^	62.08^b^	**2.29b**^**c**^	**50.3**^**b**^	**0.46**^**c**^
Kulfo	42.39^c^	121.4^c^	14.75^d^	69.61^a^	2.46^b^	36.18^d^	0.53^a^
NASPOT-12	**55.88**^**a**^	122.07^c^	17.17^c^	71.53^a^	2.93^a^	61.50^a^	0.48^bc^
NASPOT-13	47.55^b^	100.36^d^	18.25b^c^	59.71^b^	2.96^a^	50.15^b^	0.48^bc^
VITA	43.53^bc^	109.87^cd^	18.67^b^	59.22^b^	2.31^bc^	40.62^cd^	0.52^ab^
Mean	**45.45**	**126.24**	**18.22**	**62.53**	**2.57**	**46.56**	**0.49**
LSD	**5.02**	**15.07**	**1.45**	**4.92**	**0.37**	**8.26**	**0.04**
CV (%)	**19.40**	**20.9**	**13.94**	**13.78**	**25.51**	**31.09**	**14.43**

Means followed by the same letter are not statistically different from each other.

DF = Degree of freedom, TSRW = Total fresh storage root yield (ton ha^−1^), VL = Vine length (cm), SRL = Storage root length (cm), SRG = Storage root girth (mm), MSRN = Marketable storage root number, WAGB = weight of above ground fresh biomass (ton ha^−1^); HI = harvestable index (%).

### 3.3 Variance estimate for total fresh storage root yield and related variables of orange fleshed sweet potato genotypes

The combined ANOVA for total fresh storage root yield and related variables revealed that there were high significant variations (P < 0.01) among the genotypes, environment, year, Y∗E, Y∗G, and Y∗E∗G (Table 6). These significant variations indicated that the response of the genotypes were fluctuated and varied in their total fresh storage yield and change in environment and this phenomenon’s declares the presence of genotype by environmental interactions.

**Table 6:** The combined analysis of variance for the mean of total fresh storage root yield (ton ha^−1^) and yield related variables of nine orange fleshed sweet potato genotypes.

Sources of variation	DF	Mean square						
		VL	SRL	SRG	MSRN	AGBW	TSRY	HI
Environment (E)	3	11896.8∗∗∗	29.44∗∗	4050.3∗∗∗	7.02∗∗∗	1724.4∗∗∗	2477.6∗∗∗	0.16∗∗∗
Genotype (G)	8	14611.7∗∗∗	56.05∗∗∗	537.3∗∗∗	3.26∗∗∗	1462.3∗∗∗	426.0∗∗∗	0.015∗∗
Year (Y)	1	6647.5∗∗∗	15.39	2995.8∗∗∗	3.01∗∗∗	9273.7∗∗∗	5.86	0.08∗∗∗
Y∗E	3	29168∗∗∗	325.38∗∗∗	4417.6∗∗∗	17.65∗∗∗	13259.9∗∗∗	24395.7∗∗∗	0.14∗∗∗
G∗E	24	1137.0∗∗	11.19∗∗	136.2∗∗	1.08∗∗∗	723.7∗∗∗	143.3∗∗	0.01∗∗∗
G∗Y	8	816.4	5.71	109.7	0.55	462.0∗	169.3∗∗	0.01∗∗
G∗Y∗E	24	1318.6∗∗	7.24	118.8∗	0.68∗	564.9∗∗∗	160.6∗∗∗	0.01∗∗∗
Error	142	687.8	6.45	4.3	0.43	209.57	77.38	0.005

∗, ∗∗, ∗∗∗ significant @ 0.05, 0.01, and 0.001% of probability level respectively.

Df = Degree of freedom, Vl = vine length (cm), SRL = Storage root length (cm), SRG = Storage root girth (mm), MSRN= Marketable storage root number, TSRY= Total fresh storage root yield (ton ha^−1^), HI= harvestable index (%).

### 3.4 The mean performance of total fresh storage root yield (ton ha^−1^) of nine orange-fleshed sweet potato genotypes tested across eight environments

The mean total fresh storage root yields of the nine orange-fleshed sweet potato genotypes were highly variable over the eight environments. Among the genotypes, the highest mean total fresh storage root yield (55.88 ton ha^−1^) was observed from genotype NASPOT-12 and the lowest mean of total fresh storage root yield (42.4 ton ha^−1^) was recorded from the genotype Kulfo. Among the environments, the highest mean was recorded at Jimma-1 (75.74 ton ha^−1^) and the least mean was observed at Agaro-1 (19.17) (Table 7).

**Table 7:** The mean performance of total fresh storage root yield (ton ha^−1^) of nine orange-fleshed sweet potato genotypes tested across eight environments.

Genotypes	Environments								Overall mean
	Jimma-1	Agaro-1	Metu -1	Haru-1	Jimma-2	Agaro -2	Metu -2	Haru-2	
Alamura	79.77	17.97	43.62	45.07	26.59	65.24	40.07	33.02	43.92
Dilla	68.38	20.18	42.33	66.11	27.19	66.10	34.53	29.71	44.32
Guntutei	75.32	16.34	37.01	57.47	25.37	66.10	36.50	30.24	43.04
Kabode	71.86	18.06	41.81	55.88	25.72	64.44	35.62	29.86	42.91
Koka-12	59.49	24.0	46.28	79.47	28.93	66.47	31.38	28.38	45.55
Kulfo	71.73	15.83	34.67	63.70	24.67	65.96	34.15	28.48	42.40
NASPOT-12	90.10	27.86	46.06	72.37	37.84	80.10	49.61	43.08	**55.88**
NASPOT-13	84.27	15.70	24.24	78.29	28.37	75.91	40.15	33.48	**47.55**
VITA	80.78	16.58	40.27	46.52	25.94	65.77	39.85	32.60	43.54
**Mean**	**75.74**	**19.17**	**39.59**	**62.76**	**27.85**	**68.45**	**37.98**	**32.09**	**45.46**

∗ Means followed by the same letter are not significant different from each other.

### 3.5 AMMI analysis

In addition to the usual ANOVA, the ANOVA from the AMMI Model for total fresh storage root yield also detected and revealed a significant variation (p < 0.001) for both the main effect and interaction effects which indicated the existence of a wide range of variation among the genotypes, environment and their interactions.

#### 3.5.1 AMMI 1 biplot analysis

**Figure 2:**
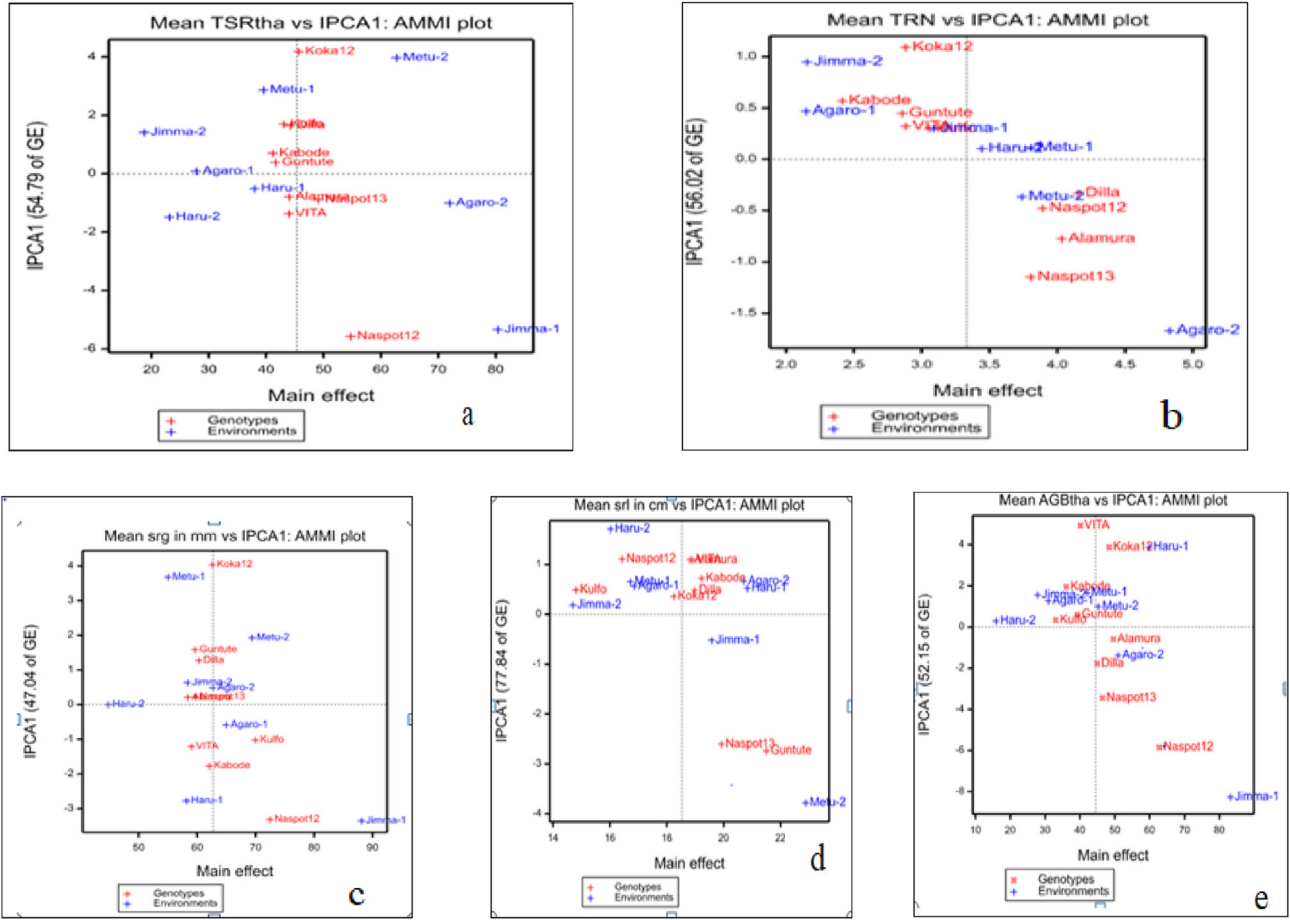
(a–e) AMMI1 biplot showing Genotype and Environmental means against IPCA1; “a” = total fresh storage root yield in ton per hectare, “b” = total storage root number, “C” = storage root girth in mm, “d” = storage root length in cm, and “e” = above ground fresh biomass weight in ton per hectare.

It is known that, the AMMI biplot analysis provides a graphical representation to close the information on the main effect and interaction of both the genotypes and the environments simultaneously. The AMMI 1 biplot containing the genotype and environmental means against interaction of principal component analysis one (IPCA1) score was illustrated in Figure 2(a–e). From this figure, the displacement along the abscissa revealed the differences in the main effect while the displacements along the ordinate exhibited the differences in the interaction effects. The genotypes and the environments with IPCA1 greater than zero were classified as a high yielding genotypes and the favorable environments whereas those with ICPA 1 lower than zero were considered to be lower yielding genotype and unfavorable environments (Manrique and Hermann, 2000; Yan and Tinker, 2005).

The graph revealed that, the genotypes NASPOT-12 and NASPOT-13 were resulted in an above average mean of total fresh storage root yield as they presented on the right side of the vertical lines which was the grand mean of the genotypes and environments (Figure 2a). Conversely the genotypes Guntutei, Alamura, VITA, and Dilla were resulted in yield below the grand mean because they were presented at the left side of the vertical line. Exceptionally, the genotype, Koka-12 laid very closely to the vertical line which indicated the mean yield of this genotype was similar to the overall environment mean. NASPOT-12 followed by NASPOT-13 had a higher mean total fresh root yield in favorable environments while Guntutei and Kabode resulted in lower mean total fresh storage root yield the unfavorable (Figure 2a). Regardless of their contribution to interaction effect, Dilla, Alamura, and VITA fall on same vertical line which was an ideal and showing their similarity in the mean yield. Regarding the environments, Jimma-1 and Agaro-2 had a root yields above the grand mean and they considered as a favorable environments (Figure 2a). The environments Jimma-2, Agaro-1, Haru-1, Haru-2, and Metu-1 had below average total fresh storage root yield and thus considered as unfavorable environments. The environments called Metu-1 and Haru-1 laid too close to the grand mean line and thus indicating that the genotypic yield in them represents the overall genotypic yield mean across all environments (Figure 2a).

The genotypes Dilla, NASPOT-12, Alamura, and NASPOT-13 had a higher mean total storage root number per plant in favorable environments while Kabode resulted in lower mean total storage root number per plant in unfavorable sites (Figure 2b). Regardless of their contribution to interaction effect, Guntutei and VITA fall on same vertical line which was an ideal and showing their similarity in the mean total storage root number. Regarding the environments, Agaro-2, Metu-2, Haru-2, and Metu-1 had a total storage root numbers above the grand mean and they considered as a favorable environments (Figure 2b). The environments Jimma-2 and Agaro-1 had below average total storage root number and thus considered as unfavorable environments. The environments called Jimma-1, Haru-1, and Haru-2 laid too close to the grand mean line and thus indicating that the genotypic total storage root number in them represents the overall genotypic total storage root number mean across all environments (Figure 2b).

The genotypes NASPOT-12 and Kulfo had a higher mean storage root girth in favorable environments while VITA, Alamura, Guntutei, Dilla, and Napot-13 resulted in lower mean total storage root girth in unfavorable sites (Figure 2c). Regardless of their contribution to interaction effect, Koka-12 and kabode fall on same vertical line which was an ideal and showing their similarity in the mean storage root girth. Regarding the environments, Jimma-1and Metu-2, had a storage root girth above the grand mean and they considered as a favorable environments (Figure 2c). The environments Haru-2 had below average storage root girth and thus considered as unfavorable environments. Those environments laid too close to the grand mean line indicating that the genotypic storage root girth in them represents the overall genotypic mean across all environments (Figure 2c).

Inversely storage root girth, the genotypes VITA, Alamura, Guntutei, Dilla, and Napot-13 had a higher mean storage root length in favorable environments while Kulfo resulted in lower mean total storage root length in unfavorable sites (Figure 2d). Regardless of their contribution to interaction effect, those genotypes fall on same vertical line which was an ideal and showing their similarity in the mean storage root length. Regarding the environments, those laid in the right side had a storage root length above the grand mean and they considered as favorable environments (Figure 2d). The environments the environments laid to left side had below average storage root length and thus considered as unfavorable environments. Those environments laid too close to the grand mean line indicating that the genotypic storage root lengths in them represents the overall genotypic mean across all environments (Figure 2d).

Similar to total storage root yield, the genotypes typically Naspt-12 and the likes had a higher mean above ground biomass weight in favorable environments while Kulfo resulted in lower mean above ground biomass weight in unfavorable sites (Figure 2e). Regarding the environments, those laid in the right side had above ground biomass weight above the grand mean and they considered as favorable environments (Figure 2e). The environments laid to left side had below average above ground biomass weight and thus considered as unfavorable environments. Those environments laid too close to the grand mean line indicating that the genotypic storage root lengths in them represents the overall genotypic mean across all environments (Figure 2e).

#### 3.5.2 AMMI 2 biplot analysis

**Figure 3:**
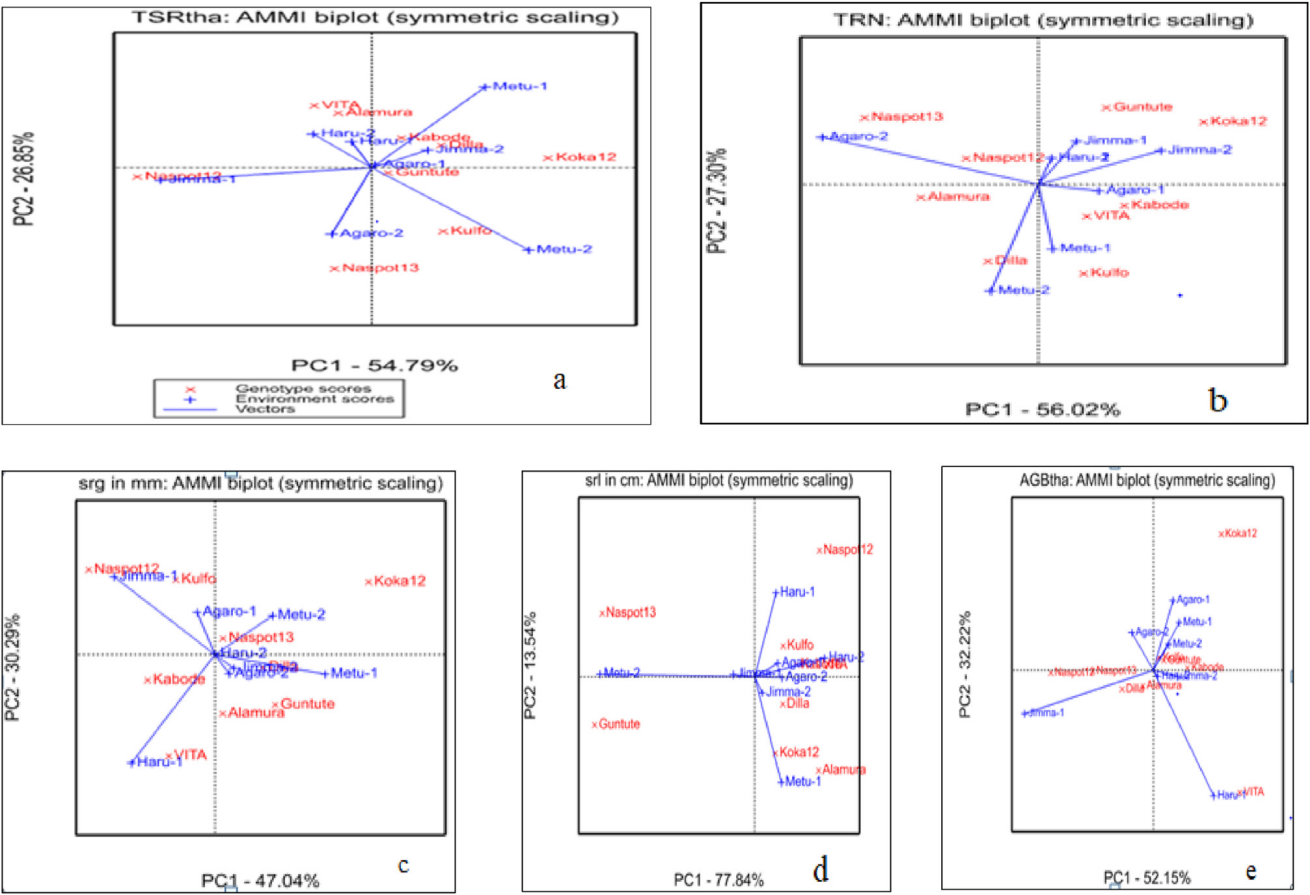
(a–e) The AMMI 2 biplot showing the PC1 versus PC2 indicating the stability of the genotypes evaluated.

The AMMI2 biplot with the IPCA1 on the X-axis and IPCA2 on the Y-axis is plotted in Figure 3(a–e). Accordingly the AMMI2 revealed that the 1^st^ interaction principal component (IPC1 or PC1) explained 55% and the 2^nd^ interaction principal component (IPC2 or PC2) explained about 27% of the genotype by environment interaction on the total fresh storage root weight ton per hectare. The two interaction principal component cumulatively explained about 82% of the genotype by environment interaction (Figure 3a).

Yan et al. (2007) reported that the closer the genotypes to the origin are more stable and the farthest genotypes from the origin are the more unstable. In addition to this, the closer the genotypes to the given vector of any environment is more adaptive to specific environment and the farthest genotypes to the given of any environment is less adaptive to that specific environment. Based on these facts the genotype Koka-12, NASPOT-13, Alamura, and VITA were far apart from the biplot origin and the environmental vectors which indicated that the genotypes were the more responsive and largely contributed to the interaction component and thus considered as specifically adapted genotypes (Figure 3a). On the other side the genotypes Kulfo, NASPOT-12, Guntutei, Kabode, and Dilla were the least contributors to the interaction components as they were located near to the biplot origin and thus indicated their wider adaptability ranges in total fresh storage root weight per hectare (Figure 3a).

The case of the adaptability of the genotypes in environments in which they were evaluated; the genotypes Kulfo was adapted to Metu-2; the genotypes NASPOT-12 was adapted to Jimma-1, and the genotypes NASPOT-13 was adapted to Agaro-2 (Figure 3a). The same interpretation was depicted for each yield related traits of orange-fleshed sweet potato: total storage root number per plant (Figure 3b), storage root girth (Figure 3c), storage root length (Figure 3d), and Above ground Biomass weight in tone per hectare (Figure 3e). In line with these facts, Tekalign (2007) reported that there were significant variations among the genotypes regarding the root yield and some genotypes revealed stability in Hararge area where they were evaluated. Similarly Ebem et al. (2021) reported that there the variation of the genotypes in his evaluation of 41 sweet potato genotypes in two different locations. Cadersa et al. (2022) also reported a closely result in his evaluation of 18 potato genotypes across four testing location.

### 3.6 GGE biplot analysis

**Figure 4:**
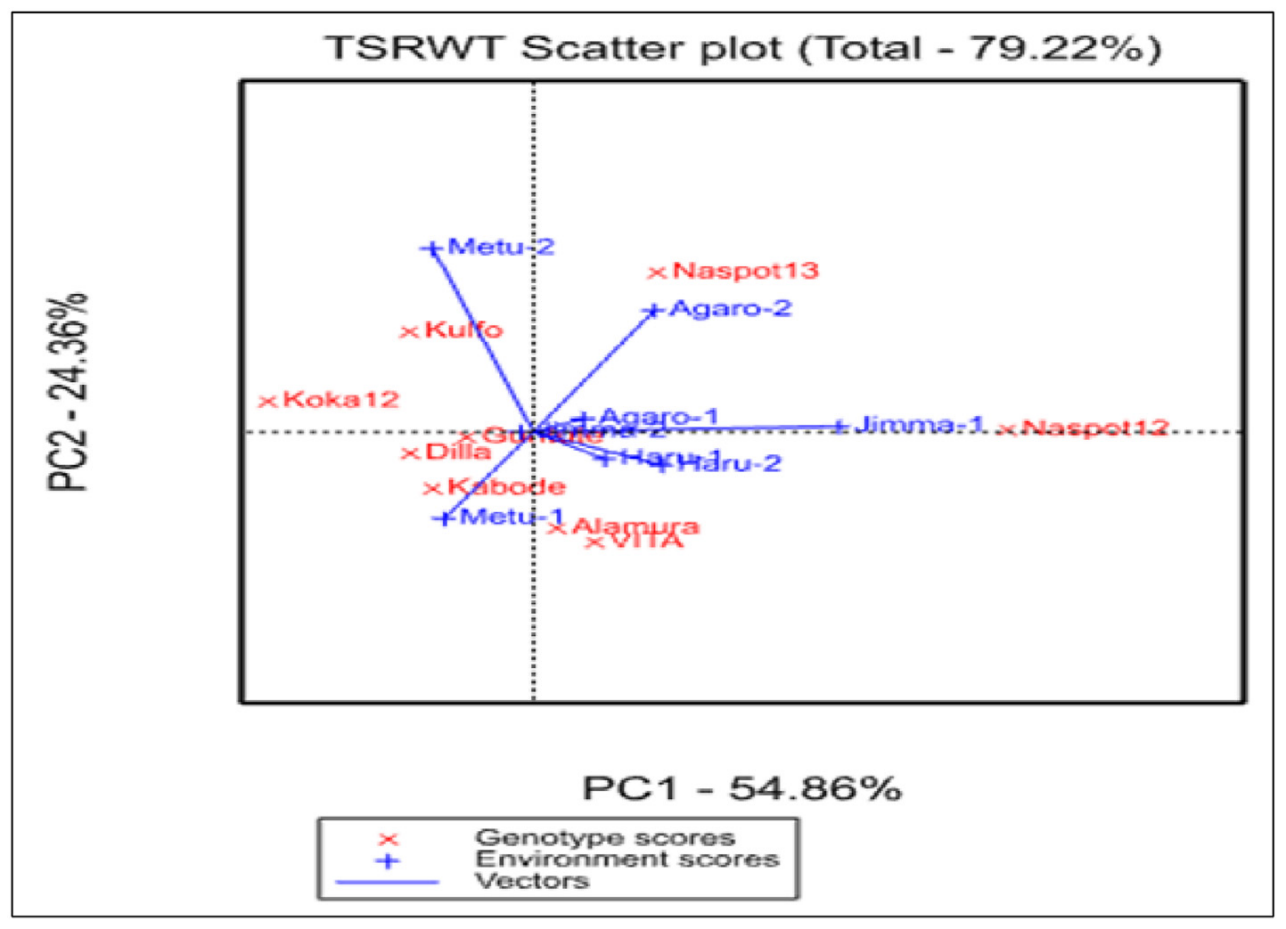
The environmental vector view of the GGE biplot showing similarities among test environments for the total fresh storage root yield of orange-fleshed sweet potato genotypes.

The first two principal components in the GGE biplot of this experiment accounted the total variations of 79% (axis 1 and axis 2) with the axis1(PC1) accounted about 55% and the axis 2(PC2) affect about 24% of variations approximately on the total fresh storage root yield of the tested orange-fleshed sweet potato genotypes. Yan and Tinker (2005) stated that the similarity and the relationship between two environments as well as the genotypes are determined by both the length of their vectors and the cosine of the angle between them. Based on these facts, the angle between Jimma-1 and Metu-1&2 is about >90° indicating there was no correlation between these environments and introducing different information about the genotypes (Figure 4). The rest of the environments had vectors with less than 90° and thus indicating that the environments were positively correlated to each other and they had close and strong relationships. Among the environments, Agaro-1 and Jimma-1 had the strong positive correlation and producing or suggesting similar information about the genotype. Such conditions are the potential to reduce the testing environments and costs. Inversely, the environment Metu-1 and Agaro-2; Metu-2 and Haru-2 had strong negative relationships and suggesting or producing different information on same genotypes. Generally environments with a cosine angle of right angle (90°) and obtuse angle (>90) are not correlated positively. This indicated that a strong crossover of GE. Farther more the distance between the two environments measures their dissimilarity in discriminating the tested genotypes. The similarity (covariance) between the two environments is determined by both the lengths of the vectors and the cosine of the angle between them. Accordingly, the Jimma-1 and Metu-2 location were the most discriminating and holds more information about the genotypes while the environments Metu-1 and Agaro-2 were less discriminating and provide medium information of the genotypes. The environments with the shortest vector from the biplot Origin were consistently non-discriminating (non-informative) provide little information on the genotypes and, there for should not be used for the test environment being they were stable (Figure 4).

#### 3.6.1 The “which won where” GGE biplot analysis

**Figure 5:**
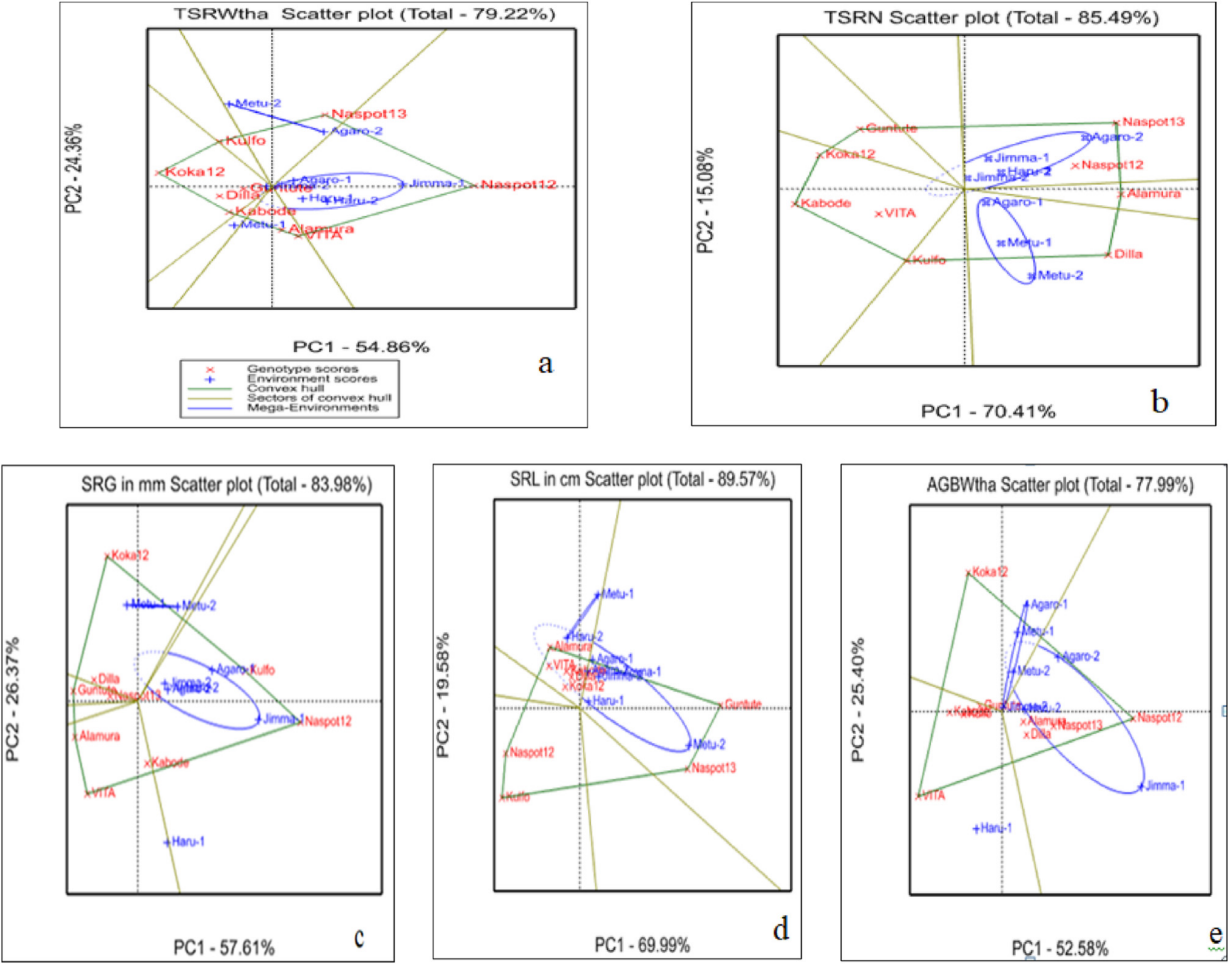
The “which-won-where” view of the GGE biplot for “a” = total fresh storage root yield in ton per hectare, “b” = total storage root number, “C” = storage root girth in mm, “d” = storage root length in cm, and “e” = above ground fresh biomass weight in ton per hectare.

To display graphically, the “which won where” a pattern of polygon views of GGE biplot for total fresh storage root yield and other related traits of the tested sweet potato genotypes was illustrated in Figure 5(a–e). The polygon was formed by connecting the vertices of the genotypes that farthest away from the biplot origin and all the genotypes were included in/on the polygon.

The GGE biplot analysis for total fresh storage root yield in ton per hectare revealed about 79% of total variation by the two principal components (Axis 1 and 2) approximately with Axis 1 accounting approximately for 55% of the total variation while Axis 2 influenced 24% of the observed variation (Figure 5a). It showed that there were three different broad environments. The 1st environments were Jimma-1, Jimma-2, Haru-1, Haru-2, and Agaro-2 with the highest storage root number of the winning genotypes NASPOT-13 and NASPOT-12 respectively. The 2nd environment Agaro-1, Metu-1 and Metu-2 were considered as suitable environments for those genotypes located in their sector with the highest winning genotype Dilla and Alamura in their number of storage roots (Figure 5a). The genotypes NASPOT-13, Dilla, Guntutei Kabode, and Kulfo were located at the vertex of the polygon and they were the winning genotypes in their located environmental sectors by their storage root number. Those genotypes located without any environment in their sectors were not a high yielding in any environment and thus they were the poorest genotypes at all or in some environments by their storage root numbers.

The GGE biplot analysis for total storage root number per plat the revealed about 86% of total variation by the two principal components (Axis 1 and 2) approximately with Axis 1 accounting approximately for 70% of the total variation while Axis 2 influenced 15% of the observed variation (Figure 5b). It showed that there were two different broad environments. The 1st environments were Jimma-1, Jimma-2, Haru-1, Haru-2, and Agaro-2 with the highest storage root number of the winning genotypes NASPOT-13 and NASPOT-12 respectively. The 2nd environment Agaro-1, Metu-1 and Metu-2 were considered as suitable environments for those genotypes located in their sector with the highest winning genotype Dilla and Alamura in their number of storage roots (Figure 5b). The genotypes NASPOT-13, Dilla, Guntutei Kabode, and Kulfo were located at the vertex of the polygon and they were the winning genotypes in their located environmental sectors by their storage root number. Those genotypes located without any environment in their sectors were not a high yielding in any environment and thus they were the poorest genotypes at all or in some environments by their storage root numbers.

The GGE biplot analysis for storage root girth in millimeter revealed about 84% of total variation by the two principal components (Axis1 and 2) approximately with Axis 1 accounting approximately for 58% of the total variation while Axis 2 approximately influenced 26% of the observed variation (Figure 5c). The analysis showed that there were three different broad environments. The 1st environments Jimma-1, Jimma-2, Agaro-1, Agaro-2 and Haru-1 with the highest storage root girth and the winning genotypes NASPOT-12. The 2nd environment were Metu-1 and Metu-2 which were considered as suitable environments for those genotypes located in their sector with the highest winning genotype koka-12 in their storage root girth (Figure 5c). The 3rd environment was haru-1 with the winning genotypes Kabode in its storage root girth (Figure 5c). The genotypes NASPOT-12, Koka-12, and Kabode were located at the vertex of the polygon and they were the winning genotypes in their located environmental sectors by their storage root girth (Figure 5c). Those genotypes located without any environment in their sectors were not a high yielding in any environment and thus they were the poorest genotypes at all or in some environments by their storage root girth (Figure 5c).

The GGE biplot analysis for storage root length in centimeter revealed about 90% of total variation by the two principal components (Axis 1 and 2) approximately with Axis 1 accounting approximately for 70% of the total variation while Axis 2 approximately influenced 20% of the observed variation (Figure 5d). The analysis revealed that there two broad environments. The 1st environments Jimma-1, Jimma-2, Agaro-1, Agaro-2, Metu-2, Haru-1 and Haru-2 with the highest storage root length and the winning genotypes NASPOT-13 and Guntutei. The 2^nd^ environment were Metu-1 and Metu-2 which were similar to storage root girth and they were considered as suitable environments for those genotypes located in their sector with the highest winning genotype Alamura in their storage root length (Figure 5d). The genotypes Alamura, NASPOT-12, NASPOT-13, Guntutei, and Kulfo were located at the vertex of the polygon and they were the winning genotypes in their located environmental sectors by their storage root length (Figure 5d). Those genotypes located without any environment in their sectors were not a high yielding in any environment and thus they were the poorest genotypes at all or in some environments by their storage root length (Figure 5d).

The GGE biplot analysis for above ground fresh biomass weight in ton per hectare of the sweet potato genotypes tested revealed about 78% of total variation by the two principal components (Axis 1 and 2) approximately with Axis 1 accounting approximately for 53% of the total variation while Axis 2 approximately influenced 25% of the observed variation (Figure 5e). The analysis also revealed that there were three broad environments. The 1st environments Jimma-1, Agaro-2, Metu-2, and Haru-2 which had the highest above ground fresh biomass weight with the winning genotypes NASPOT-13 and NASPOT-12. The 2nd environment was Agaro-1, Metu-1, Metu-2, and Jimma-2. They were considered as suitable environments for those genotypes located in their sector by their above ground fresh biomass weight (Figure 5e). The 3rd environment was Haru-1 with the winning genotypes VITA in its above ground fresh biomass weight (Figure 5e). The genotypes NASPOT-12, Koka-12, and Kulfo were located at the vertex of the polygon and they were the winning genotypes in their located environmental sectors by their above ground fresh biomass weight (Figure 5e). Those genotypes located without any environment in their sectors were not a high yielding in any environment and thus they were the poorest genotypes at all or in some environments by their above ground fresh biomass weight (Figure 5e).

#### 3.6.2 Genotype comparison by GGE biplot analysis

**Figure 6:**
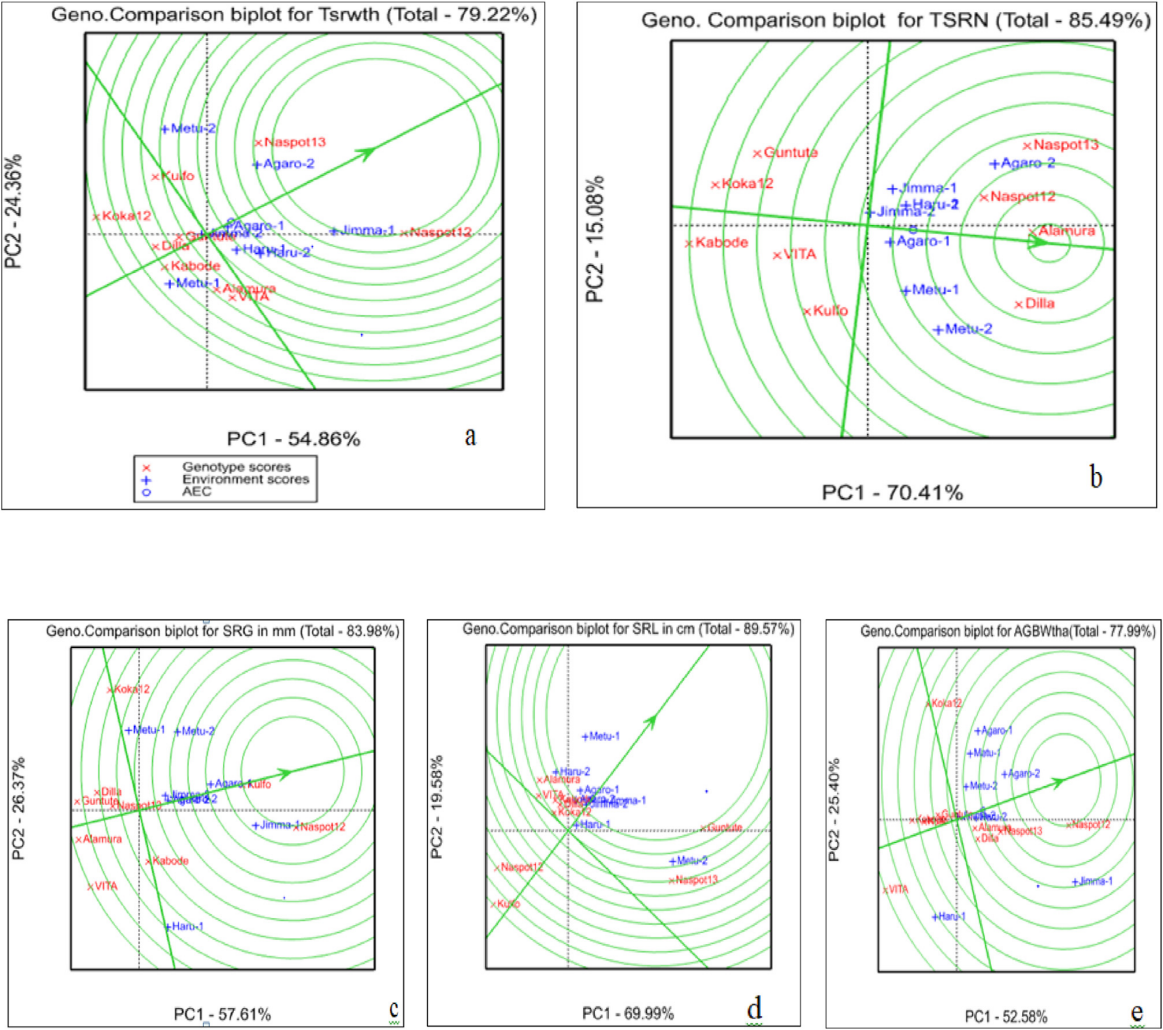
The “Genotypes comparison” view of the GGE biplot for “a” = total fresh storage root yield in ton per hectare, “b” = total storage root number, “C” = storage root girth in mm, “d” = storage root length in cm, and “e” = above ground fresh biomass weight in ton per hectare.

An ideal genotype should have both high mean performance and high stability across environments (Yan and Tinker 2006). Hence; Figure 6(a–e) defined an “ideal” genotype (the center of the concentric circles) to be a point on the AEA which was “absolutely stable” in the positive direction and had a vector length equal to the longest vectors of the genotypes on the positive side of AEA which showed the highest mean performance. Based on this fact, the genotype located closest to the center of concentric circles (“ideal”) genotype were more desirable than others. Therefore, the genotype NASPOT-12 was more desirable relative other genotypes while the genotype Koka-12 was, of course, the poorest genotype because it was consistently poor in its total fresh storage root weight ton per hectare (Figure 6a). This figure also illustrates an important concept regarding “stability”. The term “high stability” is meaning full if and only if associated with mean performance. The tested genotype is highly “stable” does not mean that the genotype was good; it only means that the relative performance of the genotype was consistent across the environment. Therefore, “stable” genotypes are desirable only when they have high mean performances.

The genotype Alamura was more desirable relative other genotypes while the genotype Kabode was, of course, the poorest genotype because it was consistently poor in its total storage root number per plant (Figure 6b), the genotype Kulfo followed by NASPOT-12 was more desirable relative other genotypes while the genotype VITA was, of course, the poorest genotype because it was consistently poor in its storage root girth in mm (Figure 6c), the genotype Guntutei was more desirable relative other genotypes while the genotype Kulfo was, of course, the poorest genotype because it was consistently poor in its storage root length in cm (Figure 6d), and the genotype NASPOT-12 followed by NASPOT-13 was more desirable relative other genotypes while the genotype VITA was, of course, the poorest genotype because it was consistently poor in its fresh above ground fresh biomass weight ton per hectare (Figure 6e).

#### 3.6.3 Environmental comparison by GGE biplot analysis

**Figure 7:**
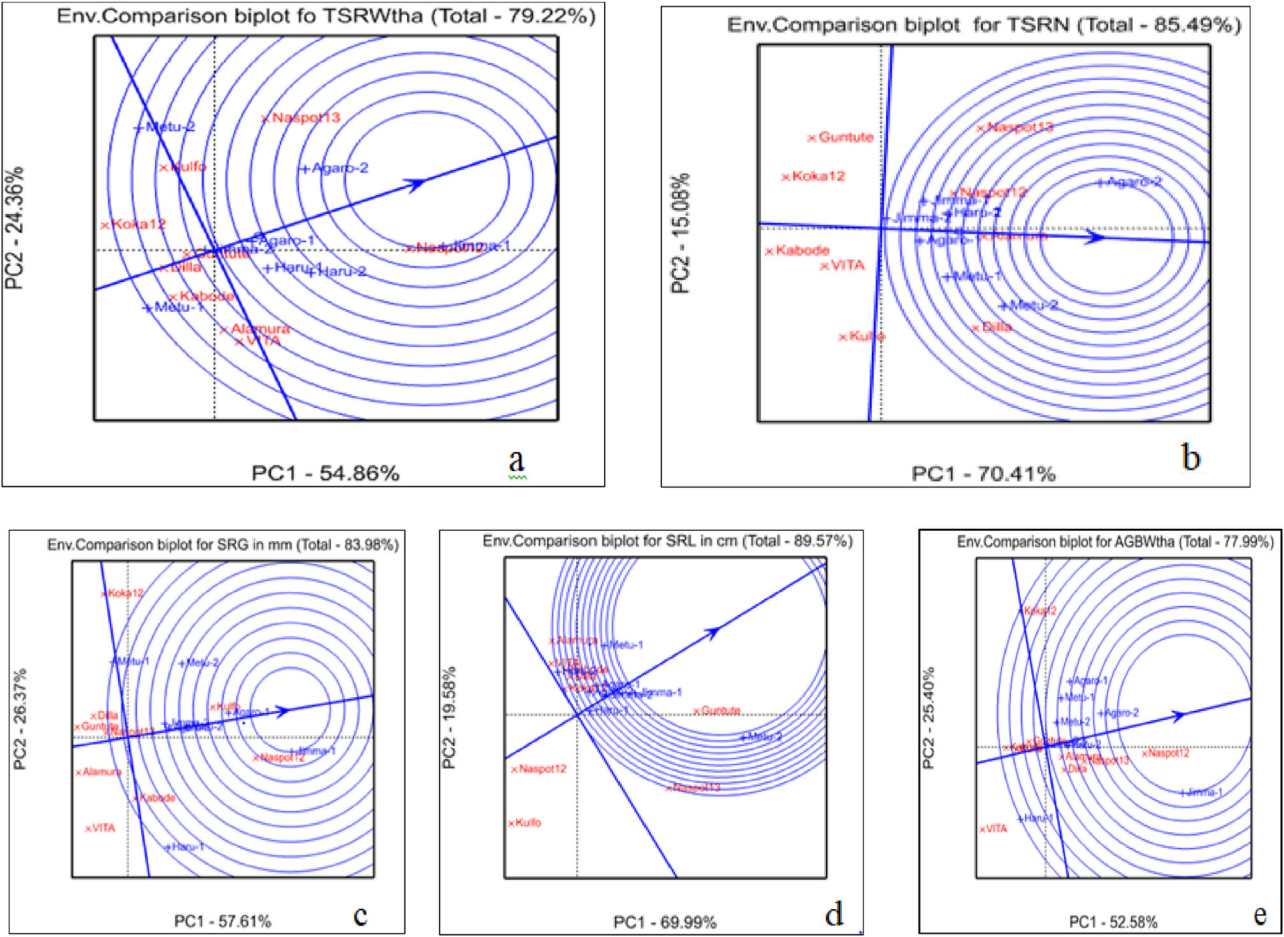
The “Environmental comparison” view of the GGE biplot for “a” = total fresh storage root yield in ton per hectare, “b” = total storage root number, “C” = storage root girth in mm, “d” = storage root length in cm, and “e” = above ground biomass weight in ton per hectare.

The line that passes through the average environment and the biplot origin is called Average Environment Axis (AEA). A test environment that has smaller angle with the AEA is more representative of the other test environment. Based this fact, the environment Jimma-1 was the most representative followed by Agaro-2 whereas Metu-1 and Metu-2 were the least representative in the total fresh storage root yield of orange-fleshed sweet potato genotypes (Figure 7a). The environment Jimma-1 was both the representative and a discriminating hence; it is a good test environment for selecting generally adapted genotypes. The environment Metu-2 was discriminating but non-representative hence it is useful for testing specially adapted genotypes if the target environment may be divided in to mega environments. The non-discriminating test environments were those with very short vectors were less useful as they provide few discriminating information about the genotypes. These were Agaro-1, Jimma-2, and Haru-1 environments (Figure 7a).

The environment Agaro-2 was the most representative whereas Metu-1and Metu-2 were the least representative in the total storage root number per plant of orange-fleshed sweet potato genotypes tested (Figure 7b). The environment Agaro-2 was both the representative and a discriminating hence; it is a good test environment for selecting generally adapted genotypes for the total storage root numbers per plant. The environment Metu-2 was discrimination but non-representative hence it is useful for testing specially adapted genotypes if the target environment may be divided in to mega environments. The non-discriminating test environments were less useful as they provide few discriminating information about the genotypes on their total storage root numbers per plant. These were Agaro-1 and Jimma-2 environments (Figure 7b).

The environment Jimma-1 was the most representative followed by Agaro-1 whereas Metu-1and Haru-1were the least representatives in the storage root girth of orange-fleshed sweet potato genotypes tested (Figure 7c). The environment Jimma-1 was both the representative and a discriminating followed by Agaro-1, hence; it is a good test environment for selecting generally adapted genotypes for the storage root girth. The environment Metu-1 was discriminating but non-representative hence it is useful for testing specially adapted genotypes if the target environment may be divided in to mega environments. The non-discriminating test environments were less useful as they provide few discriminating information about the genotypes on their storage root girth. These were Jimma-2 and Agaro -2 environments (Figure 7c).

The environment Jimma-1 was the most representative followed by Metu-2 in the storage roots length of orange-fleshed sweet potato genotypes (Figure 7d). The environment Jimma-1 was both the representative and a discriminating followed by Agaro-1, hence; it is a good test environment for selecting generally adapted genotypes for the storage root length (Figure 7d).

The environment Jimma-1 was the most representative followed by Agaro-2 whereas Haru-1 were the least representative in the above ground fresh biomass weight ton per hectare of orange-fleshed sweet potato genotypes (Figure 7e). The environment Jimma-1 was both the representative and a discriminating hence; it is a good test environment for selecting generally adapted genotypes for the above ground fresh biomass weight. The environment Haru-1 was discriminating but non-representative hence it is useful for testing specially adapted genotypes if the target environment may be divided in to mega environments. The non-discriminating test environments were less useful as they provide few discriminating information about the genotypes on their above ground fresh biomass weight. These were Haru-2 and Jimma-2 environments (Figure 7e).

#### 3.6.4 Genotype ranking by GGE biplot analysis

**Figure 8:**
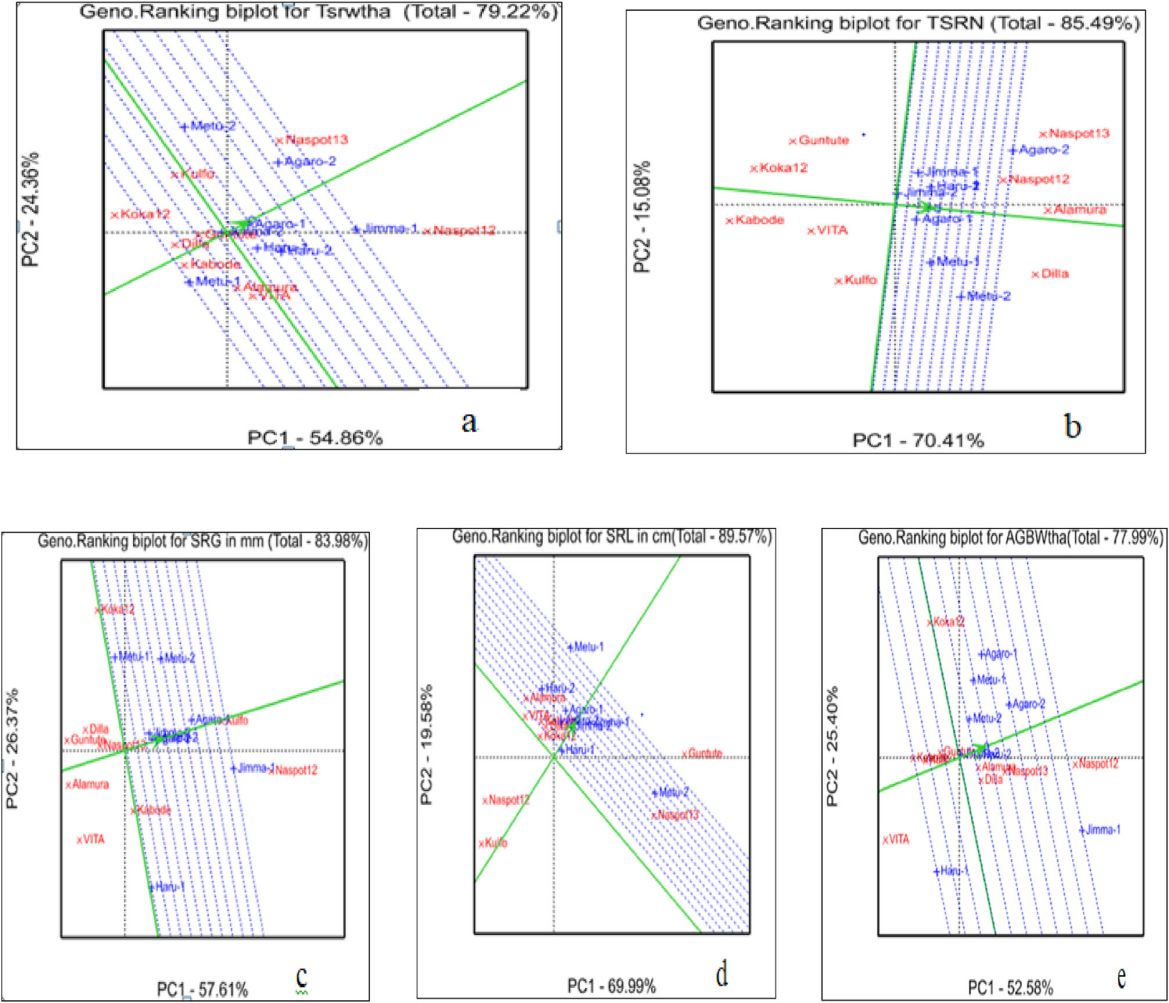
The “Genotypes Ranking” view of the GGE biplot for “a” = total fresh storage root yield in ton per hectare, “b” = total storage root number, “C” = storage root girth in mm, “d” = storage root length in cm, and “e” = above ground biomass weight in ton per hectare.

The genotypes tested in eight location or eight growing season were ranked by the GGE biplot analysis based on their yield and yield related traits (Figure 8(a–e)). Accordingly, the genotypes NASPOT-12 and NASPOT-13 were the superiors while the genotypes Kulfo and Kabode were the least in their total fresh storage root yield ton per hectare across the tested environments (Figure 8a). The genotypes Alamura, NASPOT-13, Dilla and NASPOT-12 were the superiors while the genotypes Kabode and Koka-12 were the least in their total storage root number per plant across the tested environments (Figure 8b). The genotype NASPOT-12 was the superior while the genotypes Alamura, VITA, and Guntutei were the least in their storage root girth across the tested environments (Figure 8c). The genotype Guntutei was the superior while the genotypes Kulfo was the least in their storage root length across the tested environments (Figure 8d). The genotype NASPOT-12 was the superior while the genotypes VITA was the least in their above ground biomass weight ton per hectare across the tested environments (Figure 8e).

#### 3.6.5 Environmental ranking by GGE biplot analysis

**Figure 9:**
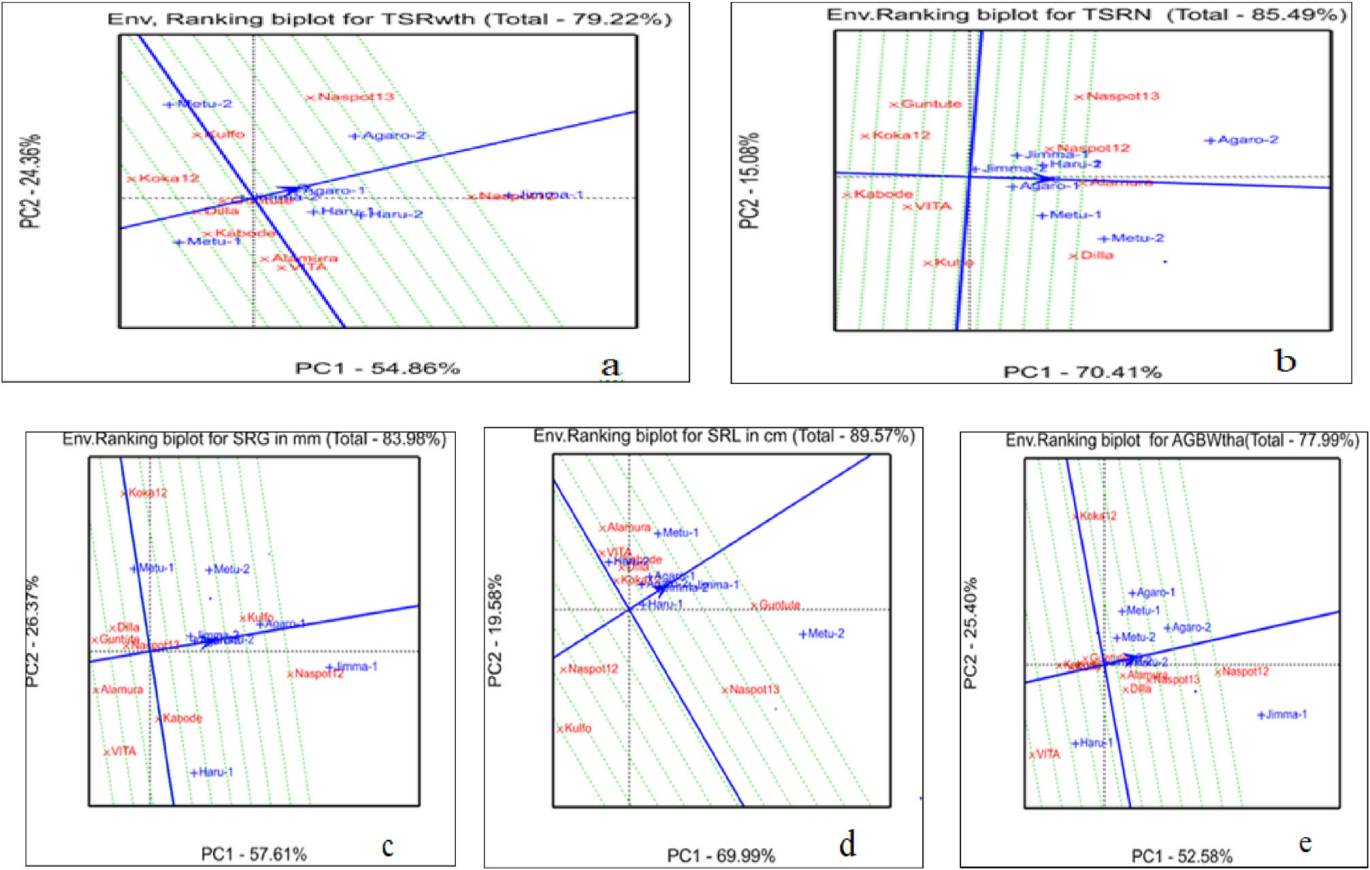
The “Genotypes Ranking” view of the GGE biplot for “a” = total fresh storage root yield in ton per hectare, “b” = total storage root number, “C” = storage root girth in mm, “d” = storage root length in cm, and “e” = above ground biomass weight in ton per hectare.

The line that passes through the average environment and the biplot origin is called Average Environment Axis (AEA). The eight locations or eight growing season in which nine orange-fleshed sweet potato genotypes tested were ranked by the GGE biplot analysis based on their response to yield and yield related traits of the genotypes (Fig-ure 9(a–e)). Accordingly, the growing season Jimma-1/Jimma 1st year and Agaro-2/Agaro 2nd year were responded as the best while growing season Metu-1/Metu 1st year was responded poorly to the total fresh storage root weight in ton per hectare of the genotypes (Figure 9a). The growing season Agaro-2/Agaro 2nd year and Metu-2/Metu 2nd year were responded as the best while growing season Jimma-2 was responded poorly to the total storage root number per plant of the genotypes tested (Figure 9b). The growing season Jimma-1/Jimma 1st year and Agaro-1/Agaro 1st year were responded as the best while growing season Metu-1/Metu 1st year was responded poorly to the storage root girth of the genotypes (Figure 9c). The growing season Metu-2 was responded as the best while growing season Haru-2 was responded poorly to the storage root length of the genotypes (Figure 9d). The growing season Jimma-1/Jimma 1st was responded as the best while growing season Haru-1/Haru 1st year was responded poorly to the above ground fresh biomass weight in ton per hectare of the genotypes (Figure 9e).

## 4 Conclusions

Yield and yield related variables were varies among Orange-fleshed sweet potato genotypes and across environments. The environments were highly variable due to their climatic/edaphic factors. The variability in the performance of the genotypes across environments made a difficulty to identify superior and stable genotypes for all locations. The genotypes NASPOT-12 was the most outperformed of all the evaluated genotypes. Some of the genotypes were more responsive and largely contributed to the interaction and thus considered as specifically adapted genotypes. Above all, the genotype NASPOT-12 was responded well in most of the environments hence, recommended genotype for multipurpose advantage.

## Declarations

### Author contribution statement

Getachew Etana Gemechu: Conceived and designed the experiments; Performed the experiments; Analyzed and interpreted the data; Contributed reagents, materials, analysis tools or data; Wrote the paper.

Tewodros Mulualem Beyene: Analyzed and interpreted the data; Contributed reagents, materials, analysis tools or data; Wrote the paper.

Neim Semman Abadura: Performed the experiments; Contributed reagents, materials, analysis tools or data; Wrote the paper.

### Funding statement

This work was supported by the Ethiopian institute of agricultural research through government fund.

### Data availability statement

The data that has been used is confidential.

### Declaration of interest’s statement

The authors declare no competing interests.

### Additional information

No additional information is available for this paper.
